# Novel preclinical gastroenteropancreatic neuroendocrine neoplasia models demonstrate the feasibility of mutation-based targeted therapy

**DOI:** 10.1007/s13402-022-00727-z

**Published:** 2022-10-21

**Authors:** Fabrice Viol, Bence Sipos, Martina Fahl, Till S. Clauditz, Tania Amin, Malte Kriegs, Maike Nieser, Jakob R. Izbicki, Samuel Huber, Ansgar W. Lohse, Jörg Schrader

**Affiliations:** 1 I. Department of Medicine, University Medical Center Hamburg-Eppendorf, Martinistrasse 52, 20246 Hamburg, Germany; 2 Internal Medicine VIII, University Hospital Tübingen, Tübingen, Germany; 3 Institute of Pathology, University Medical Center Hamburg-Eppendorf, Hamburg, Germany; 4 Laboratory of Radiobiology & Experimental Radiation Oncology, UCCH Kinomics Core Facility, Hubertus Wald Tumorzentrum, University Cancer Center Hamburg (UCCH), University Medical Center Hamburg-Eppendorf, Hamburg, Germany; 5 Center for Genomics and Transcriptomics, Tübingen, Germany; 6 Department for General, Visceral and Thoracic Surgery, University Medical Center Hamburg-Eppendorf, Hamburg, Germany; 7 Department of Medicine, Klinikum Nordfriesland, Husum, Germany

**Keywords:** Neuroendocrine neoplasm, Neuroendocrine tumor, Neuroendocrine carcinoma, Mutation based targeted therapy, DAXX, ARID1A

## Abstract

**Purpose:**

Gastroenteropancreatic neuroendocrine neoplasms (GEP-NEN) form a rare and remarkably heterogeneous group of tumors. Therefore, establishing personalized therapies is eminently challenging. To achieve progress in preclinical drug development, there is an urgent need for relevant tumor models.

**Methods:**

We successfully established three gastroenteropancreatic neuroendocrine tumor (GEP-NET) cell lines (NT-18P, NT-18LM, NT-36) and two gastroenteropancreatic neuroendocrine carcinoma (GEP-NEC) cell lines (NT-32 and NT-38). We performed a comprehensive characterization of morphology, NET differentiation, proliferation and intracellular signaling pathways of these five cell lines and, in addition, of the NT-3 GEP-NET cell line. Additionally, we conducted panel sequencing to identify genomic alterations suitable for mutation-based targeted therapy.

**Results:**

We found that the GEP-NEN cell lines exhibit a stable neuroendocrine phenotype. Functional kinome profiling revealed a higher activity of serine/threonine kinases (STK) as well as protein tyrosine kinases (PTK) in the GEP-NET cell lines NT-3 and NT-18LM compared to the GEP-NEC cell lines NT-32 and NT-38. Panel sequencing revealed a mutation in *Death Domain Associated Protein* (DAXX), sensitizing NT-18LM to the Ataxia telangiectasia and Rad3 related (ATR) inhibitor Berzosertib, and a mutation in *AT-Rich Interaction Domain 1A* (ARID1A), sensitizing NT-38 to the Aurora kinase A inhibitor Alisertib. Small interfering RNA-mediated knock down of DAXX in the DAXX wild type cell line NT-3 sensitized these cells to Berzosertib.

**Conclusions:**

The newly established GEP-NET and GEP-NEC cell lines represent comprehensive preclinical in vitro models suitable to decipher GEP-NEN biology and pathogenesis. Additionally, we present the first results of a GEP-NEN-specific mutation-based targeted therapy. These findings open up new potentialities for personalized therapies in GEP-NEN.

**Supplementary Information:**

The online version contains supplementary material available at 10.1007/s13402-022-00727-z.

## Introduction

Neuroendocrine neoplasms (NEN) form a heterogeneous group of malignancies comprising well differentiated neuroendocrine tumors (NET) and poorly differentiated neuroendocrine carcinomas (NEC). NEN originate from neuroendocrine cells ubiquitously dispersed throughout the body, in which the gastroenteropancreatic (GEP) tract represents the major site of primary tumor development [1, 2]. Although GEP-NEN are very rare in that they account for approximately 0.5% of all newly diagnosed malignancies, their incidence and prevalence have increased significantly and more than 50% of GEP-NEN patients present with a locally advanced or systemic disease at the time of diagnosis [3, 4].

Several recent genomic investigations revealed main genes being involved in GEP-NET pathogenesis. Specifically, high occurrences of somatic mutations in the *Death Domain Associated Protein (DAXX), ATP-dependent helicase ATRX, X-linked helicase II (ATRX)* and *Multiple endocrine neoplasia type 1 (MEN1)* genes have been reported. Additionally, germline mutations have been detected in the *MEN1, CDKN1B, Von Hippel–Lindau (VHL), Neurofibromin 1 (NF1)* and *Tuberous Sclerosis Complex 2* (*TSC2)* genes [5, 6]. The epigenetic regulators DAXX and ATRX participate in chromatin remodeling and, as such, are essential for maintaining histone methylation patterns in telomeres [7]. In contrast, the more aggressive GEP-NEC are characterized by gene mutations similar to those of adenocarcinomas originating from the same organs. Here, frequently occurring mutations have been reported in the *Tumor protein P53* (*TP53), Retinoblastoma protein 1 (RB1)* and *Murine sarcoma viral oncogene homolog B* (*BRAF)* genes [8, 9]. Despite these advances, a personalized mutation-based targeted therapy in GEP-NEN has not yet been established.

Unfortunately, clinical options for systemic GEP-NEN therapy are limited. So far, mammalian target of rapamycin (mTOR) inhibitors, multi-targeted receptor tyrosine kinase inhibitors, somatostatin analogues (SSA), and peptide receptor radiotherapy (PRRT) have been approved as targeted anti proliferative therapies in GEP-NET [10]. Regarding GEP-NEC, systemic chemotherapy with platin/etoposide is the only established therapeutic option [11]. The exact limitations are outlined below.

Aberrant activation of mTOR signaling has been implicated in GEP-NEN tumorigenesis, proliferation and angiogenesis [12, 13]. The RADIANT 1–4 trials revealed a significantly prolonged progression free survival (PFS) for GEP-NEN patients treated with mTOR inhibitor Everolimus alone and in combination with SSA inhibitors [14–17]. However, lack of primary response has been observed in one third of the GEP-NEN patients while others developed secondary resistance after initial disease stabilization [18]. The same rate of treatment failure was also eminent in patients treated with the multi-kinase inhibitor Sunitinib [19, 20].

SSA are well-established for the treatment of SSTR-positive GEP-NET patients, achieving prolonged intervals of disease stabilization [21–23]. Additionally, encouraging data from the NETTER 1 trial showed long-term disease control and improvement in quality of life in GEP-NET patients after peptide receptor radionuclide therapy (PRRT) [24, 25]. However, SSA and PRRT treatment is limited to GEP-NEN patients with a high SSTR expression.

Regarding GEP-NEC, most patients do initially respond to first line chemotherapy. However, the response duration is limited to a few months and a high rate of treatment failure to second line options is observed [26]. Thus, development of a personalized therapeutic approach directly targeting critical mutations in GEP-NEN is urgently needed to improve patient care.

A major challenge in developing targeted therapies in GEP-NEN is the paucity of preclinical tumor models resembling the unique genetic and phenotypic tumor features. So far, only few GEP-NEN cell lines are available. Of these BON and QGP-1 are the most widely used cell lines. While BON is commonly regarded as a GEP-NET cell line, it is currently under discussion whether QGP-1 falls under the GEP-NET or GEP-NEC classification. Although NET marker expression is present in both the BON and QGP-1 cell lines, their genomic alterations, including mutations in tumor suppressor and oncogenes (TP53, CDKN2A/B and DPC4), do not normally occur in GEP-NET. Additionally, both cell lines exhibit an extremely high proliferation rate, hardly resembling a GEP-NET phenotype [27, 28]. Thus, there is still an open discussion whether these cell lines should be classified as NET or NEC. Regarding GEP-NEC cell lines, only a limited number of publications are available.

To overcome this limitation, we have generated three GEP-NET and two GEP-NEC cell lines. Panel sequencing not only identified typical genomic alterations in the *DAXX and MEN1* genes in the GEP-NET cell lines and in *RB1* and *TP53* in the GEP-NEC cell lines. Furthermore, we show the feasibility of a mutation-based targeted therapy. Overall, our models provide a valuable research resource for both GEP-NET and GEP-NEC.

## Methods

### Patients and cell lines

Newly established GEP-NET cell lines NT-18P, NT-18LM and NT-36 were generated from a G3 pancreatic NET (PanNET) primary tumor (NT-18P), a liver metastasis (NT-18LM) and a local recurrence 12 months after initial surgery and chemotherapy treatment (NT-36) of a 57-year-old male patient. Besides the confirmed chromogranin A (CgA) and synaptophysin (SYP) expression, histopathological examination revealed Ki-67-based proliferative indexes of up to 70%. The newly established cell line NT-32 was generated from a large cell neuroendocrine carcinoma of the pancreas of a 70-year-old female patient. Histopathology revealed the presence of CgA and SYP and a Ki-67-based proliferative index of 70%. Finally, cell line NT-38 was established from a duodenal small cell neuroendocrine carcinoma of a 78-year-old female patient. Here, histopathological examination revealed expression of SYP, while CgA expression was absent. The Ki-67-based proliferative index was 70%. The use of human tissue for the generation of permanent cell cultures was approved by the local ethical review board and informed consent from the patients was obtained prior to surgery (Ärztekammer Hamburg, PV 3548). The present study was conducted in accordance with the Declaration of Helsinki.

### Primary cell culture

Primary cell culture was performed as previously described [27]. Briefly, tumor tissue was subjected to further processing immediately after surgical resection. After mincing the tissue with a scissors and scalpel, the resulting fragments were digested with collagenase for 20 min at 37 °C. Subsequently, the digested cell suspension was strained, fresh medium was added and centrifugation was performed at 400 g for 5 min. The primary mixed cell suspension was cultured in RPMI-1640 medium supplemented with 10% FCS, penicillin/streptomycin, EGF (20 ng/ml, PeproTech) and FGF2 (10 ng/ml; PeproTech). Contaminating fibroblasts were eliminated by low adherent culture and sequential trypsinization. While GEP-NET cells preferentially attach to collagen-coated culture plates, fibroblasts adhere to all culture substrates used. After successful cryopreservation of the pure tumor cell culture, passage numbering was started and cells from passage 15 – 45 were used for the experiments.

### Cell counting

Growth curve analysis was performed by trypan blue staining and manual cell counting. Briefly, cells were seeded in 24-well-plates in medium supplemented with EGF (20 ng/ml) and FGF2 (10 ng/ml). Cells were counted in a Neubauer counting chamber on Days 1, 3, 7, 14 and 24 and doubling times were determined by exponential growth curve analysis.

### Small interfering RNA-mediated knockdown

Transfection and small interfering RNA (siRNA)-mediated knockdown of DAXX expression was performed using Lipofectamine RNAiMAX Transfection Reagent (Thermo Fisher Scientific, Massachusetts, USA) according to the manufacturer’s instructions. Briefly, 5 pmol of pre-validated, quality controlled and DAXX-specific siRNA (DAXXHSS102654 Sequence (5’ – 3’) (RNA) – GGA GUU GGA UCU CUC AGA AUU GGA U), Stealth, Thermo Fisher Scientific, Massachusetts, USA) was used for transfection. An equal concentration of scrambled RNA (scrRNA) (AM4611, Thermo Fisher Scientific, Massachusetts, USA) was used as a control.

### Immunofluorescence assay

Immunofluorescence (IFC) was used to detect CgA and SYP in GEP-NET and GEP-NEC cell lines. Additionally, IFC was performed to determine the Ki67-based proliferative index. In general, GEP-NEC and GEP-NET cells were cultured on collagen IV coated glass coverslips and fixed in 4% paraformaldehyde for 10 min at room temperature (RT). Subsequent permeabilization was performed in 0.3% Tween 20 for 5 min at RT followed by blocking in 10% BSA 0.1% Tween 20 in PBS for 1 h. Next, the coverslips were incubated with 1:100 diluted primary antibodies (anti-Ki67, anti-CgA, anti-SYP; Dako Agilent, Santa Clara, USA) in a humified chamber overnight at 4 °C. Incubation of 1:400 diluted secondary antibody (anti-mouse Alexa Fluor 555, anti-mouse Alexa Fluor 488; Thermo Fisher Scientific, Massachusetts, USA) was performed for 60 min at RT. After subsequent Hoechst 33,342 (Thermo Fisher Scientific, Massachusetts, USA) incubation for 5 min at RT, the coverslips were mounted on an object slide with a drop of fluorescent mounting medium (Thermo Fisher Scientific, Massachusetts, USA). Finally, the cells were analyzed by fluorescence microscopy using a BioRevo BZ-9000 apparatus (Keyence, Neu-Isenburg, Germany) and the proliferative Ki67 index was determined by counting Ki67 positive cells.

### MTT assay

MTT analysis was performed to quantify viable tumor cells in culture and to determine IC50 values after treatment with Adavivint, Alisertib, Berzosertib, Olaparib and Trametinib (all obtained from MedChemExpress, New York, USA). For dose response analyses, cells were seeded in a 96-well plate under standard culture conditions. After overnight culture, the cells were treated with a dilution series of the various inhibitors. 48 h after the initial treatment, all inhibitors were replenished and after 120 h MTT (5 mg/ml) was added to each well, followed by an incubation for 90 min at 37 °C. Subsequently, supernatants were aspirated, and precipitated formazan dye was solubilized with DMSO. Absorbance was measured at 540 nm using a Tecan infinite F50 spectrophotometer and viable tumor cells were calculated by the mean absorbance relative to DMSO control.

### Quantitative RT-PCR

Total RNA was isolated from cell lines using a Nucleospin RNA isolation kit (Macherey Nagl, Germany) according to manufacturer’s instructions. The RNA quality was checked by spectrophotometric absorbance measurements (260/280 ratio). Subsequently, pure RNA was converted into cDNA by reversed transcription using a first strand cDNA synthesis kit according to manufacturer’s instructions (Roche, Mannheim, Germany). Pre-validated and quality controlled TaqMan® probes (*CHGA:* Hs00900370_m1, *DAXX:* Hs00985566_g1, *ISL1*: HS00158126_m1, *NEUROD1*: HS01922995_s1, *NEUROG3*: HS01875204_s1, *PDX*: HS00236830_m1, *SSTR1*: HS00265617_s1, *SSTR2:* HS00265624_s1, *SSTR3*: HS00265633_s1, *SSTR5*: HS00990407, Thermo Fisher Scientific, Massachusetts, USA) were used to assess gene expression signatures of neuroendocrine differentiation. Samples were analyzed as duplicates in a Step One Plus Real-Time PCR System (Applied Biosystems, Foster City, USA). Using comparative Ct-analyses, gene expression values were normalized to that of the housekeeping gene *GAPDH* and were expressed as fold changes.

### Panel sequencing

Coding and flanking intronic regions were enriched using a SureSelectXT Library Prep Kit (Agilent) and a CeGaT tumor panel (Agilent), and were sequenced using an Illumina NovaSeq6000 system (CeGaT GmbH Tübingen)*.* Illumina bcl2fastq2 was used to demultiplex sequencing reads. Adapter removal was performed with Skewer. The trimmed reads were mapped to the human reference genome (hg19) using the Burrows Wheeler Aligner (BWA-mem Version 0.7.2-cegat) [28]. Local realignment of reads in target areas was performed using ABRA [29] to achieve more accurate indel calling. In reference hg19, the pseudoautosomal regions (PAR) on chromosome Y have been masked (chrY:10001–2649520, chrY:59034050–59363566). This prevents reads that fall within this homologous region on chromosome X and Y from being assigned to more than one chromosome and, thus, being discarded during mapping. Reads mapping to more than one location with an identical mapping score were discarded. Read duplicates that likely result from PCR amplification were removed using samtools (Version 0.1.18) [28]. The remaining high-quality sequences were used to determine sequence variants (single nucleotide changes and small insertions/deletions). The variants were annotated based on several internal as well as external databases and on information from scientific literature.

### Functional kinome profiling

To profile kinase activity a PamStation®12 (located at the UCCH Kinomics Core Facility, Hamburg) was used as described previously [30]. To analyse serine/threonine kinases (STK) and tyrosine kinases (PTK) specific STK- as well as PTK-PamChips® were used according to the manufacturer’s instructions (PamGene International, ´s-Hertogenbosch, The Netherlands). In brief, whole cell lysates were prepared using 1 × 10^6^ cells per 100 μl M-PER Mammalian Extraction Buffer containing Halt Phosphatase Inhibitor and EDTA-free Halt Protease Inhibitor Cocktail (1:100 each; Pierce, Waltham, Massachusetts, USA). Protein quantification was performed using a bicinchoninic acid assay according to the manufacturer´s instructions (BCA; Sigma). Per array 1 µg (STK) or 5 µg (PTK) protein and 400 µM ATP were applied. Technical triplicates were analysed, and sequence-specific peptide phosphorylation was detected either in two steps (STK: first with anti-phospho-Ser/Thr antibodies during the reaction followed by detection with a secondary antibody (polyclonal swine anti-rabbit Immunoglobulin-FITC; PamGene International) or in one step (PTK) using fluorescein–labelled antibody PY20 (Exalpha, Maynard, MA, USA). Signals were recorded using a CCD camera and Evolve software (PamGene International). After quality control the final signal intensities were log2-transformed and used for further data analysis with BioNavigator software version 5.1 (PamGene International). Unless otherwise stated, data are expressed as the average signal intensity (± s. d.) of 144 (STK)/196 (PTK) peptide spots based on end levels of the phosphorylation curve. Significant differences were determined using One-way ANOVA comparisons and Dunnett’s test.

### Western blotting and densitometry

Preparation of whole cells lysates, electrophoresis and immunoblotting were performed as previously described [31]. Briefly, primary antibody incubation was conducted overnight at 4 °C after which HRP-linked secondary antibodies were incubated for 1 h at RT. We used the following primary and secondary antibodies: anti-AKT (dilution: 1:1000, #9272, Cell Signaling, Frankfurt, Germany), anti-Actin-HRP (dilution: 1:2000, SC-47778. Santa Cruz, Heidelberg, Germany), anti-active β-catenin (dilution: 1:1000, #8814, Cell Signaling, Frankfurt, Germany), anti-Cyclin-D1 (dilution 1:1000, #2978, Cell Signaling, Frankfurt, Germany), anti-Cyclin-E1 (dilution: 1:1000, #4129, Cell Signaling, Frankfurt, Germany), anti-DAXX (dilution 1:500, #4533, Cell Signaling, Frankfurt, Germany), anti-ERK (dilution: 1:1000, SC-1647, Santa Cruz, Heidelberg, Germany), anti-GAPDH-HRP (dilution: 1:2000, SC-47724, Santa Cruz, Heidelberg, Germany), anti-GSK3β (dilution: 1:1000, #5676, Cell Signaling, Frankfurt, Germany), anti-mTOR (dilution: 1:1000, #2983, Cell Signaling, Frankfurt, Germany), anti-p21 (dilution: 1:1000, #2947, Cell Signaling, Frankfurt, Germany), anti-PCNA (dilution: 1:500, SC-9857, Santa Cruz, Heidelberg, Germany), anti-PARP (dilution: 1:1000, #9542, Cell Signaling, Frankfurt, Germany), anti-p-AKT (dilution: 1:1000, #9271, Cell Signaling, Frankfurt, Germany), anti-p-ERK (dilution: 1:1000, #4695, Cell Signaling, Frankfurt, Germany), anti-p-GSK3β (dilution: 1:1000, #9336, Cell Signaling, Frankfurt, Germany), anti-p-MEK (dilution: 1:1000, #9121, Cell Signaling, Frankfurt, Germany), anti-p-mTOR (dilution: 1:1000, #2971, SC-9857, Santa Cruz, Heidelberg, Germany), Cell Signaling, Frankfurt, Germany), anti-p-SMAD2/3 (dilution: 1:1000, SC-11769, Santa Cruz, Heidelberg, Germany), anti-SMAD2/3 (dilution: 1:1000, #3102, Cell Signaling, Frankfurt, Germany), anti-SSTR2 (dilution: 1:1000, ab134152, Abcam, Cambridge, United Kingdom) and anti-SSTR5 (dilution: 1:1000, ab109495, Abcam, Cambridge, United Kingdom). Blots were developed using SuperSignal West Dura Extended Duration Substrate (Perbio Sciences, Bonn, Germany). Average densitometric band intensities were calculated using EvolutionCapt software and were normalized to the corresponding loading controls GAPDH or Actin.

### Statistical analyses

Statistical analyses were conducted using GraphPad Prism 8. Shapiro–Wilk test was used to test for normal distribution. Comparisons of parametric data of two or more groups were done using two tailed unpaired t-test or one-way ANOVA, respectively. IC50 values were determined using an inhibitor vs. normalized response (variable slope) algorithm. *P*-values < 0.05 were considered as statistically significant * *p*-value < 0.05, ** *p*-value < 0.01. *** *p*-value = 0.001. **** *p*-value < 0.0001.) Data are presented as mean ± SD and 95% confidence interval of at least three replicates.

## Results

### GEP-NEN cell lines show neuroendocrine differentiation

**Fig. 1:**
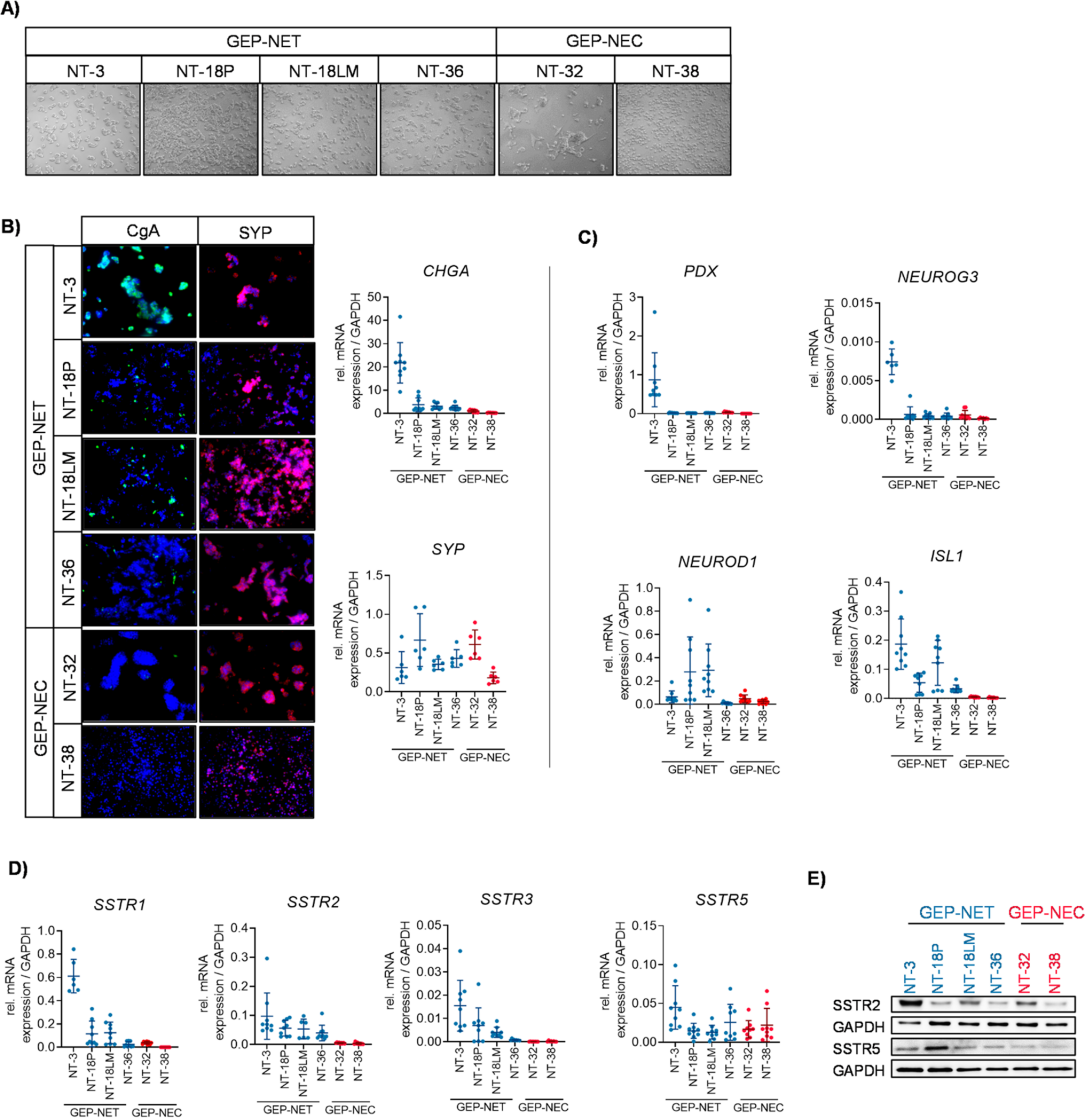
Neuroendocrine differentiation of GEP-NEN cell lines. (**A**) Representative phase contrast (PC) images. (**B**) Immunofluorescent (IFC) staining of and gene expression analysis of chromogranin A (CgA) and synaptophysin (SYP). Gene expression analyses of (**C**) neuroendocrine transcription factors and (**D**) SSTR in GEP-NET and GEP-NEC cells after 48 h of culture. Expression values were normalized to GAPDH. (**E**) Western Blot analysis of SSTR2 and SSTR5. GAPDH was used as a loading control

Fresh surgical specimens were processed by mechanical dissection and collagenase digestion. After cell straining and centrifugation, cell suspensions consisting of tumor cells and various non-neoplastic stromal cells, were transferred to uncoated culture flasks after which EGF/FGF2 supplemented medium was added. Under these conditions, small clusters of grape-like tumor cells floated in suspension, whereas fibroblasts become adherent. These fibroblasts were eliminated by 8 – 10 weeks of serial passaging and sequential trypsinization. Pure tumor cell cultures were transferred to collagen IV coated culture flasks and were cultured until a proliferating cell culture emerged. After successful cryopreservation, the morphologies and neuroendocrine phenotypes of the newly established GEP-NET cell lines NT-18P, NT-18LM and NT-36, and GEP-NEC cell lines NT-32 and NT-38 were characterized, verified and compared to the well-differentiated GEP-NET cell line NT-3 [27]. While NT-3 cells exhibit a dense spheroid-like morphology, NT-18P, NT-18LM and NT-36 cells have a more flattened appearance with occasionally dispersed and spindle shaped like cells. Particularly, the NT-36 cell line show a predominant amount of spindle shaped-like cells. The GEP-NEC cell line NT-32 displays a heterogeneous cell morphology, combining flattened spindle like cells with dense grape-like spheroids, whereas NT-38 cells appear as small dispersed cells with a lymphoid morphology (Fig. 1A). In addition to their functional activity, a high degree of neuroendocrine differentiation is one of the hallmarks of NEN. Therefore, the expression of neuroendocrine markers and neuroendocrine-related transcription factors was analyzed and compared to those of the well-differentiated NT-3 cell line. Except NT-38, expression of CgA was present in all GEP-NET cell lines, being strongest in the NT-3 cell line. Concordantly, IFC and gene expression analysis revealed expression of the neuronal marker SYP in all GEP-NET cell lines (Fig. 1B). Neuroendocrine-related transcription factors play a crucial role in islet cell differentiation during pancreas development, and their expression is maintained during neuroendocrine tumorigenesis. Thus, the expression of neuroendocrine transcription factors was analyzed. We found that * PDX* and *NEUROG3* were exclusively expressed by the well-differentiated NT-3 cell line, whereas NT-18P and NT18LM cells exhibited the highest *NEUROD1* expression among all GEP-NEN cell lines tested. *ISL1* expression was detected in all GEP-NET cell lines, whereas the GEP-NEC NT-32 and NT-38 cell lines hardly showed any *ISL1* expression (Fig. 1C). The majority of GEP-NEN are known to express somatostatin receptors (SSTR), which are exploited for nuclear imaging diagnostics and targeted therapy. Additionally, SSTR expression is known to be associated with a high degree of neuroendocrine differentiation. Here, SSTR expression was detected in every GEP-NET cell line tested, being particularly strong in the NT-3 cell line. *SSTR1/2/3* was expressed in a decreasing order by NT18P, NT-18LM and NT-36 cells, while *SSTR5* showed a similar mRNA expression among all GEP-NEN cell lines (Fig. 1D). Western blot analyses confirmed detectable protein levels of SSTR2 and SSTR5 in GEP-NEN cells. But, although *SSTR2* mRNA expression was low in NT-32, Western blot analyses revealed an increased SSTR2 protein expression (Fig. 1E). Overall, we conclude that our newly established GEP-NET and GEP-NEC cell lines are characterized by a neuroendocrine differentiation which resembles that of their tumors of origin. So far, all GEP-NEN cell lines have been successfully passaged continuously for over 36 months with no alterations in their morphology or neuroendocrine differentiation.

### GEP-NEN cell lines show proliferation that is consistent with their classification

**Fig. 2:**
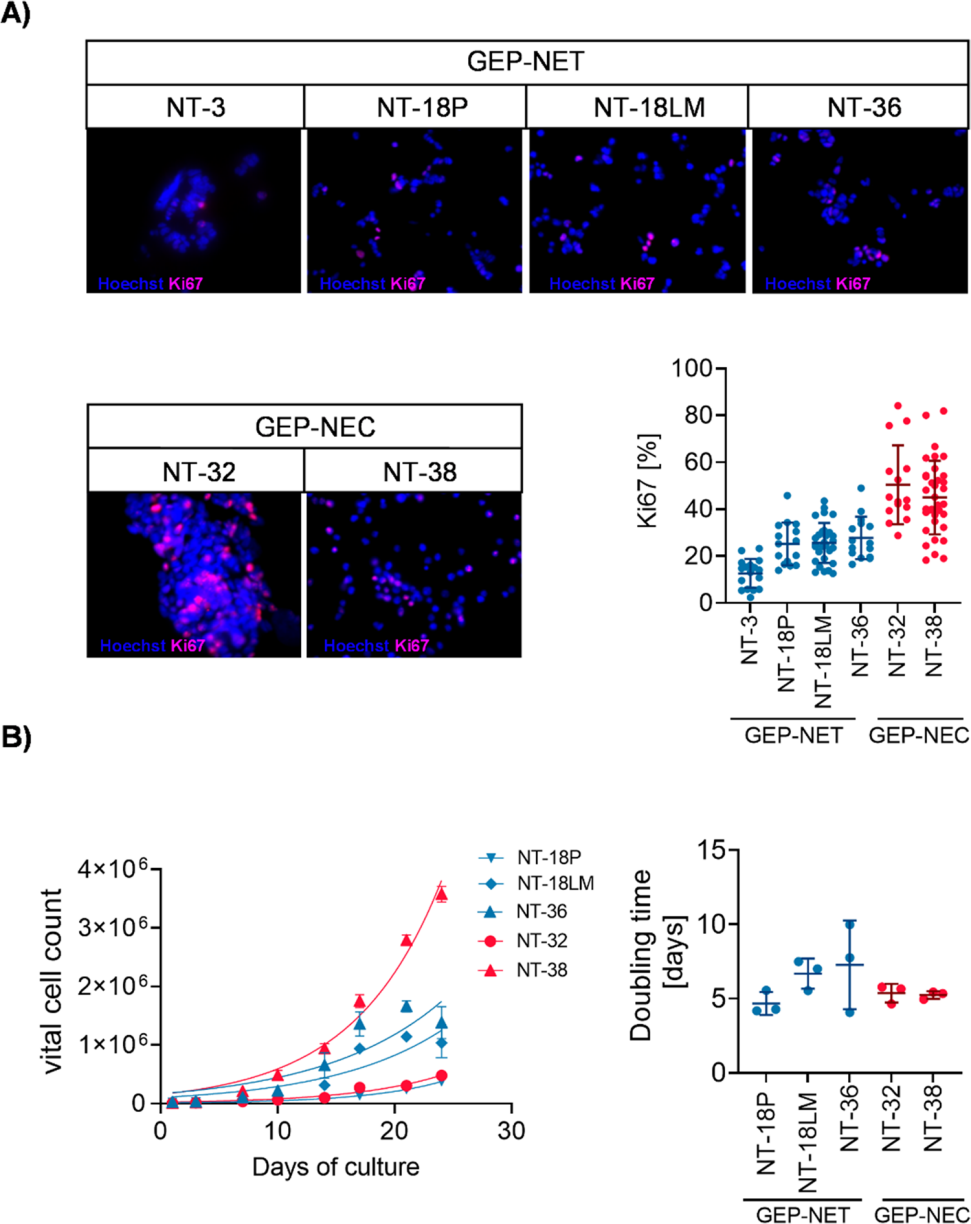
Proliferation analysis of GEP-NEN cell lines. (**A**) Immunofluorescent (IFC) staining of Ki67 in GEP-NEN cells after 48 h of culture. Ki67 proliferative indexes were calculated by the percentage of Ki67 positive cells. (**B**) Growth curves of GEP-NET and GEP-NEC cells determined in a period of 24 days. Doubling times were determined by exponential growth curve analysis

Besides the neuroendocrine differentiation, GEP-NEN are also categorized by their proliferate index. Just recently, the World Health Organization (WHO) updated GEP-NEN classification according to the grade of differentiation and Ki67 positivity. Well-differentiated GEP-NET can be subdivided into GEP-NET grade 1 (G1), grade 2 (G2) and grade 3 (G3) according to a Ki67 index (%) of < 3%, 3%—20% and > 20%, respectively. Poorly differentiated GEP-NEC exhibit a Ki67 index of > 20% and can be further subdivided into a small cell type and a large cell type. To assess the proliferative activity of our GEP-NEN cell lines, we performed immunofluorescent Ki67 staining and growth curve analyses. We found that GEP-NEC cell lines N-32 and NT-38 showed the highest Ki67 indexes with 50.40% ± 4.34% and 44.92% ± 2.65% positivity, respectively. Among the GEP-NET cell lines, NT-36 exhibited the highest Ki67 index with 27.71% ± 2.34% positivity followed by NT-18P and NT-18LM with 25.18 ± 2.35% and 25.57 ± 1.59% positivity, respectively. Consistent with a slowly proliferative G2 tumor, the well-differentiated NT-3 cell line showed the lowest Ki67 labeling index (12.58 ± 1.50%; Fig. 2A). Correspondingly, growth curve analyses revealed a doubling time of 5.21 days (95% CI: 4.80; 5.65) in the fastest growing cell line NT-38. NT-32 cells showed a doubling time of 5.35 days (95% CI: 4.73; 6.04). Although the GEP-NET cell lines NT-18P, NT-18LM and NT-36 displayed a significantly lower Ki67 positivity than the GEP-NEC cell lines NT-32 and NT-38, we observed only slightly higher doubling times of 7.09 days (95% CI: 5.43; 9.48) for NT-36 cells, 6.35 days (95% CI: 4.87; 8.42) for NT-18LM cells and 4.64 days (95% CI: 4.15; 5.20) and for NT-18P cells (Fig. 2B). All in all, our established GEP-NEN cell lines displayed a proliferative activity being consistent with their GEP-NEN classification.

### GEP-NEN cell lines exhibit a differential activation of signaling pathways

**Fig. 3:**
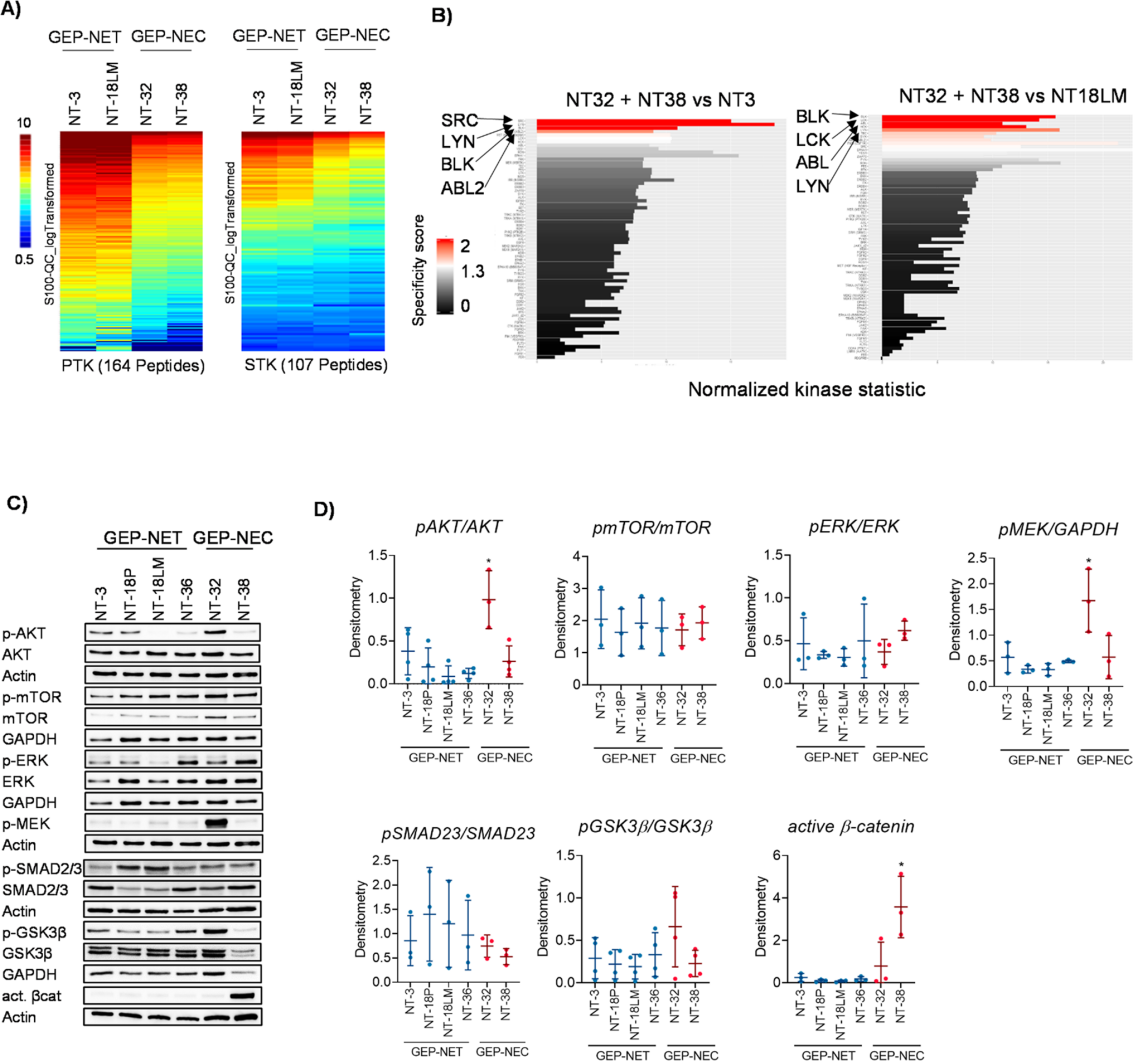
Signal transduction in GEP-NET and GEP-NEN cell lines. (**A**) Kinome analysis of NT-3, NT-18LM, NT-32 and NT-38 cells. Signal intensities of 123 phosphorylated peptides are shown as log2-transformed values in a heatmap. (**B**) Upstream PTK kinase analysis of pooled NT-32 + NT-38 vs NT-3 or NT-18LM (Normalized kinase statistic (log2) > 0: higher kinase activity in NT-3 or NT-18LM; specificity score (log2) > 1.3; white to red bars: statistically significant changes. (**C**) Western blot analysis of classical oncogenic signaling pathways in GEP-NET and GEP-NEC cell lines. GAPDH was used a loading control and representative Western blot images are shown. (**D**) Densitometric analysis of phosphorylated proteins using EvolutionCapt software. Densitometry intensities were calculated by the ratio of phosphorylated to total protein levels and subsequent normalization to GAPDH control of three biological independent experiments. * *p*-value < 0.05

In recent years, GEP-NEN research has focused on identifying aberrant activated or deactivated signal transduction pathways to achieve a better understanding of disease development and to unravel potential molecular targets for GEP-NEN therapy. As yet, only the PI3K/AKT/mTOR pathway has been identified as a promising target for GEP-NEN therapy. Therefore, we comprehensively characterized key oncogenic signaling pathways in our GEP-NEN cell lines by functional kinome prolifing and Western blotting. First, serine/threonine kinase (STK) and protein tyrosine kinase (PTK) activities in NT-3, NT-18LM, NT-32 and NT-38 cells were assessed using microarray-based functional kinomics. We observed a significantly higher number of strongly phosphorylated peptides in the GEP-NET cell lines NT-3 and NT-18LM compared to the GEP-NEC cell lines NT-32 and NT-38, i.e., the STK as well as the PTK array revealed increased overall STK and PTK activity in GEP-NET cells (Fig. 3A, Suppl. Fig. 1A, B). Analysis of upstream activated tyrosine kinases (upstream kinase analysis) indicated a high activity of Src kinase family members such as BLK, LYN, LCK in NT-3 and NT-18LM cells compared to NT-32 and NT-38 cells. Additionally, increased kinase activation of ABL signaling was detected in NT-3 and NT-18LM cells compared to NT-32 and NT-38 cells (Fig. 3B). By analyzing the STK signaling, a higher activity of AKT, ERK1/2 and PKC signaling could be observed in GEP-NET cells compared to GEP-NEC cells (Suppl. Fig. 1C). Western blotting of key oncogenic signaling pathways revealed a significantly stronger AKT phosphorylation in NT-32 cells compared to any other GEP-NEN cell line. Moreover, moderate AKT phosphorylation was detected in NT-3 and NT-18P cells, whereas hardly noticeable AKT phosphorylation was observed in NT-18LM, NT-36 and NT-38 cells. Unlike the phosphorylation levels of mTOR and ERK, which showed hardly any differences among the GEP-NEN cell lines, phosphorylation of MEK was particularly strong in the NT-32 cells. Additionally, NT-18P and NT-18LM cells showed the highest phosphorylation levels of SMAD2/3. Although phosphorylated, and thereby inactivated GSK3β levels were markedly stronger in NT-32 cells compared to NT-38 cells, NT-38 cells showed significantly more active β-catenin than NT-32 cells and the other GEP-NEN cell lines (Fig. 3C, D). Overall, we found by kinomics profiling and Western blotting that GEP-NET cells exhibit more different activated signaling pathways compared to GEP-NEC cells. Additionally, NT-32 showed a strong AKT phosphorylation and NT-38 a strong β-catenin activation.

### Panel sequencing reveals potential therapeutic targets in GEP-NEN

**Fig. 4:**
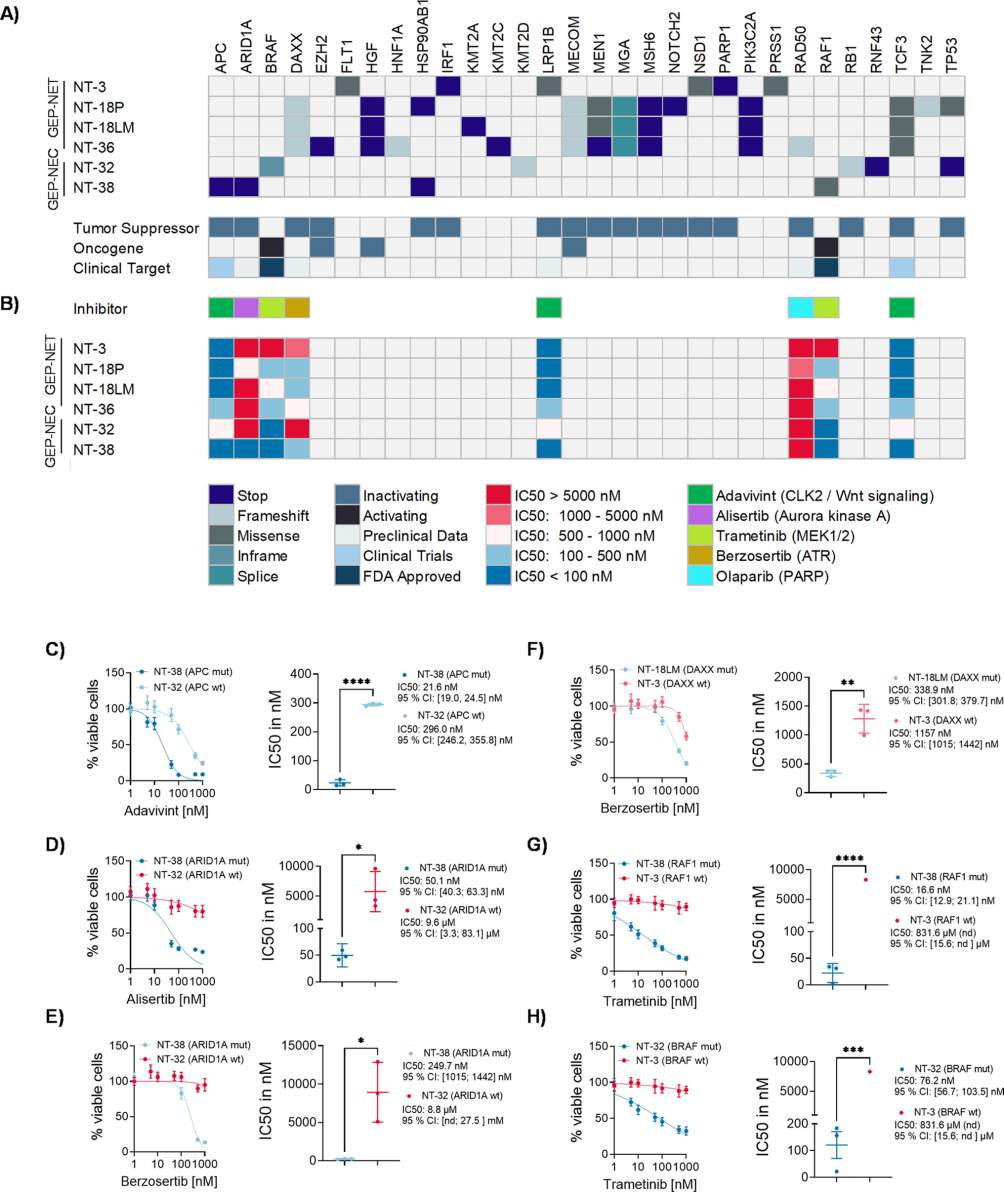
Mutational analysis of GEP-NET and GEP-NEC cell lines. (**A**) Upper panel, schematic overview of mutations in NET and NEC cell lines. Sequencing of a 700 gene panel was performed and the 30 most relevant mutations are depicted. After literature review, selected mutations were classified as potential clinical targets for inhibitors which are FDA approved in clinical trials or in a preclinical phase. (**B**) Lower panel, Schematic overview of IC50 values obtained by dose response curve analyses. (**C, D, E, F, G, H**) selected dose response curves are depicted. IC50 values were determined by inhibitor vs. normalized response (variable slope) algorithm of three biological independent experiments. IC50 values in bar charts are presented as mean and standard deviation of the mean. Additionally, 95% confidence intervals are shown. Statistical significance was calculated by an unpaired t-test. * *p*-value < 0.05, ** *p-*value < 0.01. *** *p*-value = 0.001. **** *p*-value < 0.0001

Like most cancers, GEP-NEN are characterized by specific genomic alterations. Previously published studies identified *MEN1, DAXX* and *ATRX* as the main mutated genes being apparently involved in the pathogenesis of GEP-NEN. However, further genetic modifications, especially gain of function and druggable mutations, are very rare and have not yet been elucidated. Thus, a mutation-based targeted therapy could so far not be established. Therefore, and to further characterize our established GEP-NEN cell lines, we performed sequencing of a 700 gene panel. We found that NT-3 cells harbored mutations in *LRP1B* (c.12010C > G), *NSD1* (c.2875G > A) and *PARP1* (c.826G > T). The GEP-NET cell lines NT-18P, NT-18LM and NT-36 harbored mutations in *DAXX* (c.1884dupC), *MEN1* (c.85 > T), *MSH6* (c.1095G > A), *MECOM* (c.1839delA), *PIK3C2A* (c.4694 T > A) and *TCF3* (c.302A > G). Additionally, NT-36 cells exhibited a frameshift mutation in the *RAD50* (c.2165dupA) gene. Among all GEP-NET cell lines tested, NT-18P carried a *TP53* (c.472C > A) mutation exclusively. The GEP-NEC cell line NT-32 showed a *BRAF* gain of function mutation (c.1457_1471del), whereas a frameshift mutation was detected in the *RB1* gene (c.1696-8_1699del) and stop codon mutation was detected in the *TP53* gene (c.586C > T). NT-38 cells harbored loss of function mutations in the *APC* (c.4037C > A) and *ARID1A* (c.1681C > T) genes, and a gain of function mutation was detected in the *RAF1* (c.776C > T) gene (Fig. 4A).

To explore a mutation based targeted therapy, extensive literature research was performed to identify druggable molecular targets being associated with the detected mutations. Once targets had been identified, dose response curve analyses were conducted and IC50 values were calculated. Except for NT-32, all GEP-NEN cell lines showed activating alterations in the Wnt pathway. Activation of Wnt signaling is associated with cancer stem cell renewal, proliferation and differentiation. Upon ligand binding, a complex of AXIN, GSK3β, CK1 and APC aggregates to the receptor with subsequent translocation of β-catenin to the nucleus where it interacts with T cell-specific factor (TCF)/lymphoid enhancer-binding factor (LEF) [32]. Thus, we decided to treat the cell lines with the TCF/LEF inhibitor Adavivint. We found that NT-3, NT-18P, NT-18LM, NT-36 and NT-38 cells responded to Adavivint with IC50 values < 100 nM (Fig. 4B). The *APC* mutated cell line NT-38 showed a particularly strong response to Adavivint with an IC50 of 21.6 nM (95% CI: 19.0 nM; 24.5 nM) (Fig. 4C).

A recently published study revealed that targeting Aurora kinase A induced synthetic lethality in ARID1A deficient colorectal cancer cells [33]. Here, we found that treatment of ARID1A deficient NT-38 cells with Aurora kinase A inhibitor Alisertib resulted in an IC50 of 50.1 nM (95% CI: 40.3 nM; 63.3 nM). In contrast, we found that the *ARID1A* wild type cell line NT-32 showed an IC50 of 9.6 µM (95% CI: 3.3 µM; 83.1 µM) (Fig. 4D). Additionally, it has been shown that ARID1A deficiency may lead to a synthetic lethal therapy response to Ataxia telangiectasia and Rad3 related (ATR) inhibitors in several tumor entities [34]. Here, we found that ARID1A deficient NT-38 cells responded to the ATR Inhibitor Berzosertib with an IC50 of 249.7 nM (95% CI: 214.7 nM; 288.8 nM). In contrast, NT-32 cells showed no response to Berzosertib as indicated by an IC50 of 8.8 µM (95% CI: nd; 27.5 mM) (Fig. 4E).

Moreover, previously studies revealed a significant response to ATR inhibitors in cancer cells which rely on alternative lengthening of telomeres (ALT) to overcome replicative mortality [35]. Interestingly, it has been shown that functional loss of ATRX/DAXX may be associated with an ALT phenotype in GEP-NEN [36, 37]. Thus, the *DAXX* mutated GEP-NET cell lines NT-18P, NT-18LM and NT-36 were exposed to the ATR inhibitor Berzosertib (Fig. 4B). We found that compared to the *DAXX* wild type cell line NT-3, which displayed an IC50 of 1.21 µM (95% CI: 1.03 µM; 1.61 µM), the IC50 of the *DAXX* deficient cell line NT-18LM was 338.2 nM (95% CI: 302.3 nM; 377.6 nM) (Fig. 4F).

Oncogenic activation of *RAF1* and *BRAF* was detected in NT-38 and NT-32 cells, respectively. Concordantly, administration of the MEK1/2 inhibitor Trametinib led to a significant reduction in viable cells with IC50 values of 76.2 nM (95% CI: 55.7 nM; 103.5 nM) for NT-32 cells and 16.6 nM (95% CI: 12.9 nM; 21.1 nM) for NT-38 cells, respectively (Fig. 4G, H). Although NT-36 cells showed a functional loss of *RAD50*, a therapeutic response to the PARP inhibitor Olaparib was not observed (Fig. 4B).

Taken together, we found that the newly established GEP-NET and GEP-NEC cell lines displayed characteristic GEP-NEN genetic modifications. Additionally, we found that *DAXX* deficiency rendered GEP-NET cells sensitive to ATR inhibitors, while *ARID1A* mutation in GEP-NEC cells promoted synthetic lethality upon Aurora kinase A inhibition.

### *DAXX* and* ARID1A* mutations render GEP-NEN cells sensitive to ATR and Aurora kinase A inhibitors in vitro

**Fig. 5:**
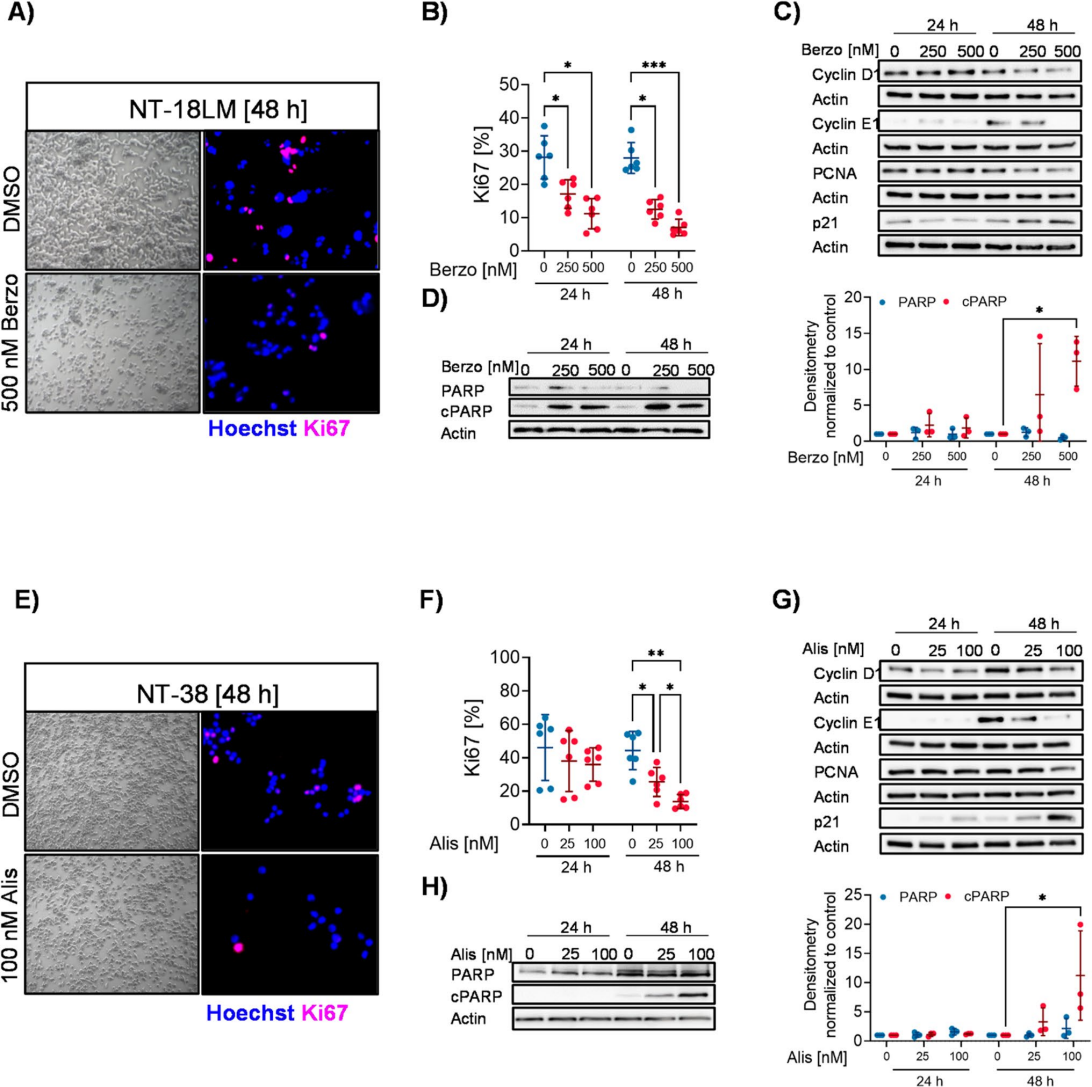
Berzosertib and Alisertib treatment in *DAXX* and *ARID1A* mutated or DAXX deficient GEP-NEN cells. **(A**) Representative phase contrast and Ki67 immunofluorescent images of NT-18LM cells treated with 500 nM Berzosertib for 48 h. (**B**) Ki67 quantification of NT-18LM cells treated with 250 nM and 500 nM Berzosertib for 24 h and 48 h. (**C**) Representative Western blot of NT-18LM cells treated with 250 nM and 500 nM Berzosertib for 24 h and 48 h. (**D**) Apoptotic activity assessed by PARP cleavage and densitometric analyses in NT-18LM cells treated with 250 nM and 500 nM Berzosertib for 24 h and 48 h. (**E**) Representative phase contrast and Ki67 immunofluorescent images of NT-38 cells treated with 100 nM Alisertib for 48 h. (**F**) Ki67 quantification of NT-38 cells treated with 25 nM and 100 nM Alisertib for 24 h and 48 h. (**G**) Representative Western blot of NT-38 cells treated with 25 nM and 100 nM Alisertib for 24 h and 48 h. (**H**) Apoptotic activity assessed by PARP cleavage and densitometric analyses in NT-38 cells treated with 25 nM and 100 nM Alisertib for 24 h and 48 h

To further characterize *DAXX* and *ARID1A* mutation as potential therapeutic targets in GEP-NEN, the effect of Berzosertib and Alisertib on proliferation and apoptosis was investigated in *DAXX* and *ARID1A* deficient cells. Additionally, siRNA-mediated expression knockdown of *DAXX* was performed in *DAXX* wild type NT-3 cells. We found that Berzosertib treatment led to a significant dose-dependent reduction in proliferation in NT-18LM cells compared to the DMSO control. Specifically, administration of 250 nM and 500 nM Berzosertib for 48 h decreased Ki67 positivity by 55.2% (*p* = 0.011) and 74.8% (*p* = 0.0001), respectively (DMSO: 27.96% ± 4.65%; 250 nM: 12.52% ± 2.91%; 500 nM: 7.04% ± 2.50%) (Fig. 5A, B). Likewise, 48 h exposure of 25 nM and 100 nM Alisertib significantly reduced the number of proliferating NT-38 cells by 42.4% (*p* = 0.0310) and 68.90% (*p* = 0.0077), respectively (48 h: DMSO: 44.33% ± 11.43%; 25 nM: 25.51% ± 8.70%; 100 nM: 13.79% ± 4.15%) (Fig. 5E, F). In addition, we found that 500 nM Berzosertib treated NT-18LM and 25 nM cells and 100 nM Alisertib treated NT-38 cells exhibited markedly lower protein levels of cyclin D1, cyclin E1, PCNA and increased protein levels of p21 after 48 h, further supporting the anti-proliferative effect of Berzosertib. We found that the p21 protein levels were particularly strong after 48 h of 100 nM Alisertib treatment (Fig. 5C, G).

Additionally, PARP cleavage was investigated to assess the effect of Berzosertib and Alisertib on apoptosis. We found that administration of 250 nM and 500 nM Berzosertib induced PARP cleavage after 24 h and 48 h, being particularly strong after 48 h. Specifically, we observed an 11.1-fold higher PARP cleavage in 500 nM Berzosertib treated NT-18LM cells compared to that in DMSO control cells (Fig. 5D). For Alisertib treated NT-38 cells, apoptosis was detected only after 48 h of Alisertib treatment, indicated by the presence of an 11.2 fold higher PARP cleavage in 100 nM Alisertib treated NT-38 cells compared to DMSO control cells (Fig. 5H).

**Fig. 6:**
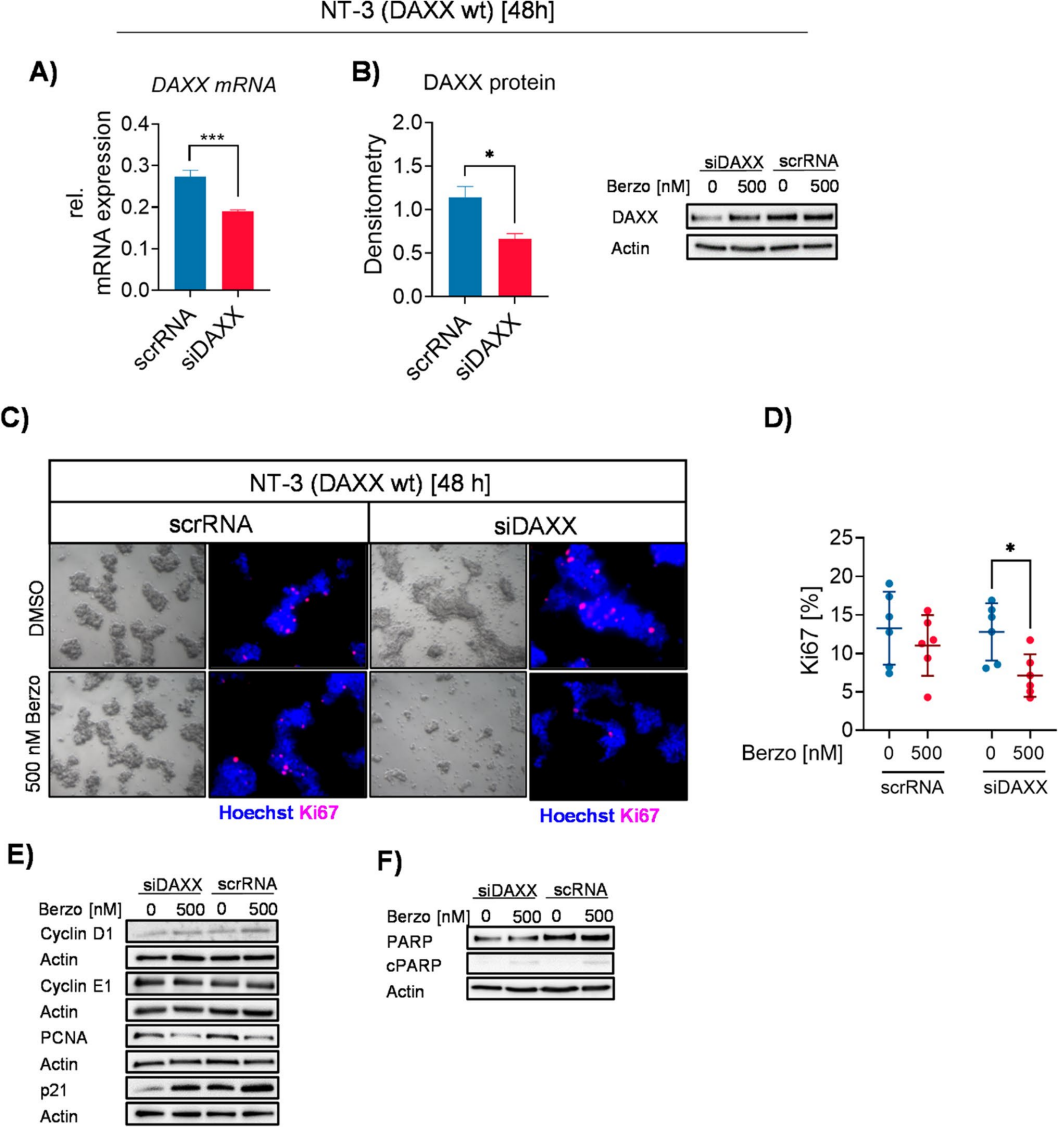
ATR inhibitor Berzosertib response in siRNA-mediated DAXX deficient NT-3 cells. (**A**) qPCR and (**B**) Western blot assessment of siRNA-mediated knockdown in DAXX wild type cell line NT-3. (**C**) Representative phase contrast and Ki67 immunofluorescent images of NT-3 cells transfected with 5 pmol of DAXX-specific siRNA (siDAXX) or scrambled RNA (scrRNA) control. Cells were treated with DMSO or 500 nM Berzosertib for 48 h. (**D**) Ki67 quantification of transfected (siDAXX, scrRNA) NT-3 cells treated with 500 nM Berzosertib or DMSO control for 48 h. (**E)** Representative Western blot of transfected NT-3 cells treated 500 nM Berzosertib for 48 h. (**F**) Representative Western blot of PARP cleavage of transfected (siDAXX, scrRNA) NT-3 cells. Actin was used as loading control. * *p*-value < 0.05, ** *p*-value < 0.01. *** *p*-value = 0.001

To further assess the role of DAXX deficiency in ATR inhibitor responsiveness, an siRNA mediated knockdown of DAXX was performed in the *DAXX* wild type NET cell line NT-3. Quantitative RT-PCR revealed a siRNA-mediated reduction in DAXX expression of 30.89% (Fig. 6A). Concordantly, densitometric Western blot analyses confirmed a DAXX knockdown of 41.65% in protein level (Fig. 6B). Phase contrast microscopy revealed a stressed morphology in NT-3 cells upon DAXX knockdown. Additionally, DAXX deficient and Berzosertib treated NT-3 cells exhibited a reduction in cell number and cell colony size compared to scrambled control and DAXX deficient and untreated NT-3 cells (Fig. 5J). While DAXX knockdown in untreated NT-3 cells had no impact on proliferation and expression of Cyclin D1, Cyclin E1, PCNA and p21 proteins, siRNA-mediated DAXX abrogation sensitized NT-3 cells to Berzosertib as indicated by increased p21 protein levels and a decrease of 44.29% (*p* = 0.0109) in proliferating cells (scrRNA DMSO: 13.29 ± 4.7, scrRNA 500 nM: 11.04 ± 3.95, siDAXX DMSO: 12.80 ± 3.72, siDAXX 500 nM: 7.13 ± 2.77) (Fig. 5J, K, L). However, functional abrogation of DAXX did not sensitize NT-3 cells towards apoptosis upon Berzosertib treatment (Fig. 5M).

## Discussion

GEP-NEN represents a minority among cancers of the digestive system and their rarity impedes the development of novel treatment regimens through large clinical studies. In addition, there is still a scarcity of reliable preclinical in vitro tumor models. Especially, to unravel molecular mechanisms underlying the unique tumor biology of GEP-NEN, well-characterized GEP-NET and GEP-NEC cell lines are urgently needed. To address this problem, we generated five novel GEP-NEN cell lines and showed that personalized molecular targeted therapies based on genetic profiling are feasible.

Establishing reliable GEP-NEN cell lines has remained exceptionally difficult, mainly due to their low proliferative behavior and unresolved issues regarding optimal culture conditions. We successfully generated three GEP-NET and two GEP-NEC cell lines. All GEP-NET cell lines exhibited a neuroendocrine phenotype as confirmed by the presence of CgA and SYP, and expression of the NET-specific transcription factors *ISL1* and *NEUROD1*. Additionally, these cell lines express *SSTR2* and *SSTR5,* further indicating their neuroendocrine origin. Regarding the GEP-NEC cell lines, NT-38 cells lack expression of CgA, whereas both NT-32 and NT-38 cells express SYP. Importantly, all GEP-NEN cell lines maintained their neuroendocrine differentiation and morphology over 30 passages, rendering them into stable and continuous cell lines. Although NT-32 cells exhibit a heterogenous morphology, this morphology was consistent over the passages. Based on intra-tumoral heterogeneity, it is conceivable that some cells in culture respond differently to growth factors present in the culture medium. With a Ki67 proliferation index of 20%—30%, the newly established GEP-NET cell lines exhibit an unambiguously lower proliferation rate than that of the most commonly used PanNET cell lines BON and QGP-1. Despite the expression of SYP as a marker of neuroendocrine differentiation in both cell lines, they exhibit atypical GEP-NET mutations (e.g. *Ras, p53*). Additionally, BON and QGP-1 exhibit a Ki-67-based proliferation index of > 90%, which hardly resembles the clinical nature of GEP-NET. Consequently, our cell lines represent a better model to investigate the role of GEP-NET specific mutations, thereby achieving a better understanding of GEP-NET biology. While there are additional GEP-NET cell lines (KRJ-I, GOT1, P-STS, L-STS and H-STS) available, recent studies have shown that only GOT1 and P-STS exhibit a true neuroendocrine phenotype. A lymphoblastoid origin was postulated for the other three cell lines, further underpinning the challenging intricacy of GEP-NET cell line generation [38]. Regarding GEP-NEC, three well-characterized cell lines have previously been established. DUE1 is derived from liver metastasis of a large cell gastroesophageal carcinoma and DUE2 from lymph node metastasis of a large cell colorectal carcinoma [39]. More recently, the first small cell NEC cell line, DUE3, has been established from a NEC of the anal canal [40]. DUE1 and DUE2 cells exhibit a neuroendocrine phenotype, a strong tumorigenicity and Ki67-based proliferation indexes of 70% (DUE1) and 30–40% (DUE2). In comparison, NT-32 and NT-38 showed a slightly lower Ki67 proliferation index.

Using kinomics analyses, we found a significantly higher frequency of tyrosine-phosphorylated peptides in the GEP-NET cells NT-3 and NT-18LM compared to that in the GEP-NEC cells NT-32 and NT-38. In particular, we found that members of the SrcB subfamily (LCK, BLK and LYN) and the ABL kinase were strongly activated in the GEP-NET cells NT-3 and NT-18LM. Recently, upregulation of LCK was reported in primary and metastatic PanNET samples, while LCK expression was absent in normal islet cells [41]. Intriguingly, Di Florio et al*.* observed a diminished adhesion and migration of PanNET cells after pharmacological inhibition of Src kinase family members [42]. Additionally, increased Src activation was observed in a GEP-NET cancer stem cell (CSC) subpopulation and Src inhibition by Dasatinib selectively reduced CSC growth, confirming Src as a relevant therapeutic target in GEP-NET [43]. Regarding ABL kinase activation, a comparative study showed a response of GEP-NET cell lines towards Imatinib independently of the expression of c-kit, suggesting a potential of ABL activity in Imatinib responsive GEP-NET cells [44]. However, previous clinical studies failed to show benefit of Imatinib treatment in GEP-NET patients, possibly due to the fact that only a few patients were enrolled. [45]. Collectively and in line with the literature, our findings indicate a potential of Src as a means of targeting GEP-NET.

Genomic alterations resulting in an aberrantly activated growth of tumor cells, have been widely used as therapeutic targets in many cancers. Whereas, GEP-NET lack a classical oncogene addiction, GEP-NEC oncogene addiction has so far not been well studied. Thus, establishing targeted treatments remains challenging. Using our newly established GEP-NEN cell lines, we identified potentially druggable genomic modifications paving the way for the design of novel treatment strategies for GEP-NET and GEP-NEC patients. Specifically, we found that NT-18P, NT-18LM and NT-36 cells harbor deactivating *DAXX* mutations. DAXX was originally identified as a Fas death receptor binding protein mediating JNK-induced apoptosis [46, 47]. However, recent studies have shown that DAXX participates in chromatin remodeling by forming a H3.3-specfic deposition complex with ATRX particularly enriched in telomeric repeats [48, 49]. A recent investigation of a multi-institutional cohort of 561 PanNET patients revealed a functional loss of DAXX/ATRX in 30% of the cases [50]. Although the exact function of a DAXX/ATRX complex at telomeric repeats remains elusive, functional loss of DAXX/ATRX is often associated with ALT, suggesting a regulatory role in telomere biology [50–52]. Interestingly, Flynn et al. found that particularly ALT-positive cancer cells are hypersensitive to pharmacological inhibition of ATR, one of the main DNA damage checkpoint-activating kinases in human cells [35, 53]. Berzosertib is a highly potent and selective inhibitor of ATR. Recent studies already demonstrated its efficacy in several cancers, especially in combination with other cytostatic drugs [54–56]. Treatment of our *DAXX* mutated cell line NT-18LM with the ATR inhibitor Berzosertib showed a reduction in viable cells, a reduction in proliferating cells and a significant induction of apoptosis. SiRNA-mediated knockdown of DAXX sensitized the *DAXX* wild type cell line NT-3 to ATR inhibition. However, apoptosis was not induced by Berzosertib in siDAXX transfected NT-3 cells. It is conceivable that transfected NT-3 cells develop only a partial ALT phenotype accompanied by a lower responsiveness to Berzozertib compared to *DAXX* mutated tumor cells, since siRNA-mediated DAXX knockdown resulted in only a partial reduction in DAXX expression. Even though a recently published study showed that ALT positive cells do not exhibit a general sensitivity to ATR inhibitors, we were able to demonstrate a response of DAXX mutated/knockdown cells to ATR inhibition [53]. Thus, it is also conceivable that DAXX deficient cells respond to ATR inhibitors independently of ALT. Further studies should focus on the mechanisms by which DAXX deficiency leads to ATR inhibitor responsiveness. As 30% of PanNET patients harbor a *DAXX* mutation this could be clinically developed into the first personalized therapy for GEP-NET patients.

A potential druggable genomic alteration was also found in the GEP-NEC cell line NT-38. Here, we detected a loss of function mutation in *ARID1A*. ARID1A represents a subunit of the SWI/SNF chromatin remodeling complex which is involved in regulating the accessibility of transcription factors, DNA polymerases and DNA damage repair proteins to specific regions within the genome. As a tumor suppressor, ARID1A was already identified in various cancers as being able to modulate cellular proliferation, invasion and metastasis [57]. In a previous study, loss of ARID1A was detected in 42.9% of non-functional PanNET associated with a malignant behavior and an unfavorable prognosis [58]. Intriguingly, Wu et al*.* revealed synthetic lethality in ARID1A deficient colorectal cancer cells to Aurora kinase A inhibitors. Alisertib, a specific Aurora kinase A antagonist has already been found to induce a potent reduction in tumor growth in solid as well as hematologic neoplasms [59, 60]. Concordantly, we found that ARID1A deficient NT-38 cells exhibited a strong sensitivity to the Aurora kinase A inhibitor Alisertib. Additionally, ARID1A deficient NT-38 cells showed a significant reduction in cellular viability upon ATR inhibition, including a reduced proliferation and a strong induction of apoptosis. Just recently, concordant results were reported by Williamson et al*.* in breast and colon cancer cells [61]. Unfortunately, we failed to establish ARID1A knockdown in our ARID1A wild type cell line NT-32, but further studies will be performed to investigate the role of ARID1A in GEP-NEN tumorigenesis and in GEP-NEN treatment.

We also identified *BRAF* and *RAF1* mutations in NT-32 and NT-38 cells, respectively. Both cell lines showed a response to the MEK inhibitor Trametinib, but not to the BRAFV600E-specific inhibitor RAF709. In GEP-NEC, the incidence of *BRAF* mutations is estimated to be 5%—25%. Two recent case reports of high-grade colorectal NEC showed an initial response to combined BRAF/MEK inhibition, but both experienced disease progression after a short treatment period [62–64]. Hence, the role of *BRAF* mutations in GEP-NEC proved to be more complex than expected. Our GEP-NEC cell lines represent an ideal tool to investigate MEK/RAF inhibitor treatment response.

Deregulated Wnt/β-catenin signaling has been implicated in neuroendocrine tumorigenesis, and prior studies showed antitumor properties of Wnt inhibitors in GEP-NEN cells [65–68]. Indeed, NT-38 cells harbored an *APC* mutation accompanied by increased activation β-catenin. Treatment of NT-38 cells with the Wnt inhibitor Adavivint led to a strong reduction in cellular viability in our study. Comprehensive molecular profiling of GEP-NEC identified *APC* mutations, particularly in colorectal NEC, with similar frequencies to those in colorectal adenocarcinomas. Consequently, it can be assumed that the primary site of GEP-NEC development is a determinant factor in the occurrence of this specific mutation. Whether *APC* mutations represent potential therapeutic targets is questionable as, so far, effective Wnt inhibitors have been shown to impair healthy tissue homeostasis and regeneration [69]. Therefore, further studies are needed to resolve the problems associated with targeting Wnt/beta-catenin in cancer.

Obviously, our study has limitations. First, we presented only a small number of cell lines. Thus, given the well-known heterogeneity of GEP-NEN, these cell lines do hardly capture the full spectrum of GEP-NEN biology. Secondly, we did not study how the identified mutations affect tumor biology, therapeutic response to established therapies and tumor progression. Particularly, in vivo studies of xenografted cells will enhance our knowledge in this regard. Nevertheless, establishing continuous cell lines will maintain a crucial role in cancer research, especially for mechanistic and therapeutic studies [70].

Future studies will include molecular and functional genomics to identify genes and expression profiles associated with malignant progression and therapy resistance. Another great opportunity of the new cell lines is to perform unbiased drug screening aiming to identify novel treatment concepts for GEP-NEN. Additionally, we will verify the treatment efficacy of Berzosertib and Alisertib in *DAXX-* and *ARID1A*-mutated in vivo xenograft models and investigate how ATR and Aurora kinase A inhibition reduces growth in *DAXX-* and *ARID1A-*mutated tumors on a molecular basis.

## Conclusion

We successfully generated three GEP-NET and two GEP-NEC cell lines proving them as a valuable resource to further investigate GEP-NET and GEP-NEC. These models allowed us to assess novel therapeutic strategies. In particular, pharmacological inhibition of ATR and Aurora kinase A in DAXX and ARID1A deficient cells may represent a promising therapeutic strategy in GEP-NEN patients.

## Supplementary Information

Below is the link to the electronic supplementary material.

Supplementary Figure 1 Box plots summarizing the log2-transformed signal intensities of (**A**) PTK array and (**B**) STK array. Upstream STK kinase analysis of pooled NT-32 + NT-38 vs NT-3 (**C**) or NT-18LM (**D**) (Normalized kinase statistic (log2) > 0: higher kinase activity in NT-3 or NT-18LM; specificity score (log2) > 1.3; white to red bars: statistically significant changes. (DOCX 395 kb)

## Data Availability

The sequencing data of the current study and the presented new cell lines are available from the corresponding author upon reasonable request. Cell lines will be available through contact with our research group under a Material Transfer Agreement (MTA).

## Abbreviations

*ATR*: Ataxia telangiectasia and Rad3 related
*ARID1A*: AT-Rich Interaction Domain 1A
*DAXX*: Death Domain Associated Protein
*CgA*: Chromogranin A
*GEP-NEN*: Gastroenteropancreatic neuroendocrine neoplasm
*GEP-NET*: Gastroenteropancreatic neuroendocrine tumor
*GEP-NEC*: Gastroenteropancreatic neuroendocrine carcinoma
*mTOR*: Mammalian target of rapamycin
*PFS*: Progression free survival
*PRRT*: Peptide receptor radiotherapy
*PTK*: Tyrosine kinase
*SSA*: Somatostatin analogues
*STK*: Serine/threonine kinase
*scrRNA*: Scrambled RNA
*siRNA*: Small interfering RNA
*SYP*: Synaptophysin
