# Supplementary figures and images for: Novel preclinical gastroenteropancreatic neuroendocrine neoplasia models demonstrate the feasibility of mutation-based targeted therapy
Source: Cell Oncol (Dordr). 2022 Oct 21;45(6):1401–19. doi: 10.1007/s13402-022-00727-z (PMC9747820; doi:10.1007/s13402-022-00727-z)

**Supplementary Figure 1 Functional kinome profiling of GEP-NEN cell lines**


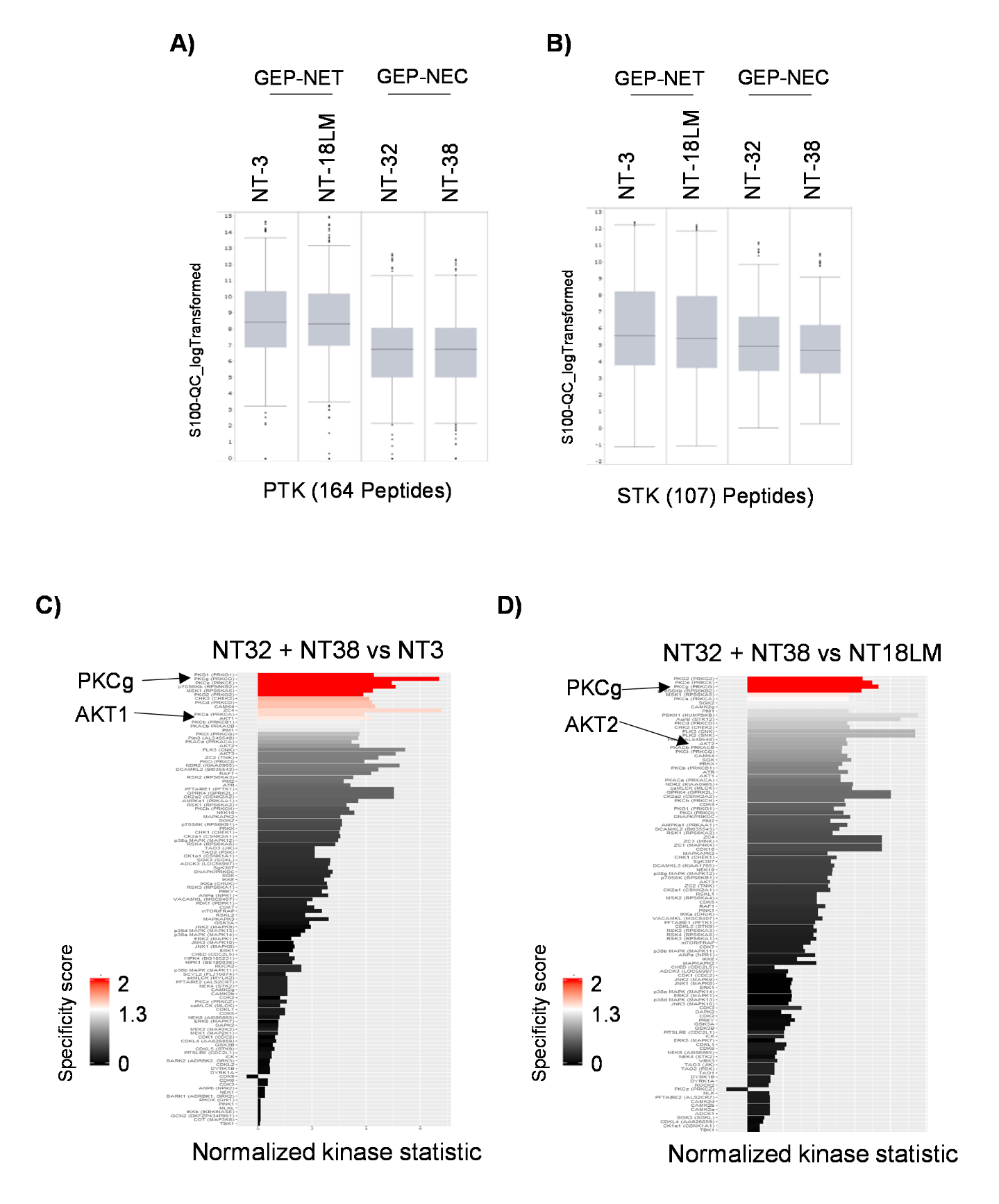

Supplement: Supplementary file 1 — Supplementary Figure 1 Box plots summarizing the log2-transformed signal intensities of (A) PTK array and (B) STK array. Upstream STK kinase analysis of pooled NT-32 + NT-38 vs NT-3 (C) or NT-18LM (D) (Normalized kinase statistic (log2) > 0: higher kinase activity in NT-3 or NT-18LM; specificity score (log2) > 1.3; white to red bars: statistically significant changes. (DOCX 395 kb) [file 13402_2022_727_MOESM1_ESM.docx]
